# Strukturmerkmale und Voraussetzungen zur ambulanten Implantation von implantierbaren Defibrillatoren, Geräten zur kardialen Resynchronisation und Ereignisrekordern

**DOI:** 10.1007/s00399-021-00764-5

**Published:** 2021-05-12

**Authors:** Thomas M. Helms, Ralph Bosch, Claudius Hansen, Cord Willhöft, Bettina Zippel-Schultz, Christoph Karle, Jörg Otto Schwab

**Affiliations:** 1 Peri Cor Arbeitsgruppe Kardiologie/Ass. UCSF, Hamburg, Deutschland; 2 Deutsche Stiftung für chronisch Kranke, Fürth, Deutschland; 3 Cardio Centrum Ludwigsburg, Ludwigsburg, Deutschland; 4 Herz- & Gefäßzentrum am Krankenhaus Neu-Bethlehem, Göttingen, Deutschland; 5 Fieldfisher, Deutschland LLP, München, Deutschland; 6 Deutsche Stiftung für chronisch Kranke, Berlin, Deutschland; 7 PraeMedicum Praxis für Diagnostik, Künzelsau, Deutschland; 8 Kardiologie, Beta Klinik Bonn, Bonn, Deutschland

**Keywords:** Ereignisrekorder, Ambulante Implantation, Qualitätssicherung, Holistisches Konzept, Individualisierte Therapie, Outpatient implantation, Event recorders, Quality management, Individualized therapy, Holistic concept

## Abstract

Die Möglichkeiten der ambulanten Implantation von Defibrillatoren, Geräten zur kardialen Resynchronisation und Ereignisrekordern („cardiac implantable electronic devices“, CIEDs) gewinnen zunehmend an Bedeutung. In Deutschland existieren aktuell vereinzelte Möglichkeiten zur ambulanten Implantation. Es fehlt allerdings an einheitlichen, anerkannten und verbindlichen Qualitätskriterien sowie an Grundzügen vertraglicher Gestaltungen. Der vorliegende Artikel stellt einen Einblick in die aktuellen Vertragskonstellationen zur ambulanten Operation bereit und definiert ein erstes, holistisches Qualitätskonzept für ambulante Implantationen von CIEDs. Im Zentrum steht das Ziel, einen Diskurs in der Fachgesellschaft anzustoßen, um ein abgestimmtes, verbindliches Qualitätskonzept zu definieren. Dieses sollte als Grundlage für zukünftige Leistungen der ambulanten Implantation dienen, die Leistungen vergleichbar machen und einen Beitrag zum langfristigen Nachweis der Evidenz leisten.

## Abstract (en)

The possibility of outpatient implantation of defibrillators, devices for cardiac resynchronization, and event recorders (collectively called cardiac implantable electronic devices, CIEDs) is becoming increasingly important. In Germany, only a few options for outpatient implantation are currently realized. Furthermore, there is a lack of uniform, recognized, and binding quality criteria. This article provides insight into the current contract constellations for outpatient surgery and defines a first, holistic quality concept for outpatient implantations of CIEDs. The present works aims to initiate a discourse in the specialist society in order to define a coordinated, binding quality concept. Then, this should serve as the basis for future outpatient implantation services, enabling comparability and to contribute long-term evidence.

Die Individualisierung der Device-Therapie durch eine patientenorientierte Entscheidung zur ambulanten Implantation von implantierbaren Kardioverter-Defibrillatoren (ICD), Geräten zur Resynchronisationstherapie (CRT-P/D) sowie Ereignisrekordern (ILR) ist in Deutschland nicht einheitlich geregelt. Die nachweislich positiven Effekte der Device-Therapie haben zu einem Anstieg von Implantationen geführt, wobei sich die stationäre und die ambulante Implantation gegenüberstehen. Mehrere Studien geben Hinweise darauf, dass sich die ambulante Implantation mehr an den individuellen Bedürfnissen des Patienten orientiert und kosteneffizient erbracht werden kann. In Deutschland existieren aktuell nur vereinzelte und nicht flächendeckende Möglichkeiten, ICD-, CRT-Systeme sowie ILR ambulant zu implantieren. Es erfolgt zwar bereits eine Verlagerung der Implantation in den ambulanten Sektor. Allerdings wird die Möglichkeit der ambulanten Implantation aufgrund fehlender Vergütungsstrukturen bei Weitem nicht ausgenutzt. Außerdem fehlen einheitliche Kriterien, die die Qualität der ambulanten Implantation absichern, eine Vergleichbarkeit über alle Sektoren hinweg ermöglichen und eine Datenerhebung generieren, aus der die weitere Entwicklung der Implantationen im ambulanten Sektor optimiert werden kann. Im Folgenden wird der Status quo der ambulanten Operation von implantierten kardialen Rhythmusdevices („cardiac implantable electronic devices“, CIEDs) in Deutschland und in internationalen Studien beleuchtet sowie ein ganzheitliches Qualitätskonzept vorgeschlagen.

## Definitionen

### Ambulant vs. stationär

Ein maßgeblicher Grundsatz des Leistungsrechts im Sozialgesetzbuch V ist „ambulant vor stationär“. Nach § 39 Abs. 1 S. 2 SGB V haben gesetzlich Krankenversicherte Anspruch auf vollstationäre oder stationsäquivalente Behandlung durch ein nach § 108 zugelassenes Krankenhaus, sofern das Behandlungsziel nicht durch teilstationäre, vor- und nachstationäre oder ambulante Behandlung erreicht werden kann.^1^ Nach diesem abgestuften System darf die aufwändige Leistungsform der vollstationären Behandlung nur gewährt werden, wenn sie erforderlich ist und das Behandlungsziel nicht durch ambulante Leistungen erreicht werden kann.^2^ Im Gegenzug bedeutet dies, dass eine vollstationäre Behandlung nur dann erforderlich ist, wenn die notwendige medizinische Versorgung ausschließlich mit den besonderen Mitteln des Krankenhauses verfolgt werden kann und eine ambulante ärztliche Versorgung nicht ausreicht, um eine Krankheit zu erkennen, zu heilen, ihre Verschlimmerung zu verhüten oder Krankheitsbeschwerden zu lindern.^3^ Dieses Prinzip ist dabei eine spezielle Ausformung des Wirtschaftlichkeitsprinzips nach § 12 Abs. 1 SGB V: Durch den Vorrang ambulant vor stationär sollen Anreize für medizinisch nicht erforderliche und teure vollstationäre Maßnahmen abgebaut und damit zur Kostensenkung beigetragen werden^4^, mithin also ein Vorrang der preisgünstigen ambulanten Behandlung.^5^ Die Erforderlichkeit einer vollstationären Krankenhausbehandlung muss in einer einzelfallbezogenen Gesamtbetrachtung beurteilt werden. Ob einem Versicherten vollstationäre Krankenhausbehandlung zu gewähren ist, richtet sich allein nach den medizinischen Erfordernissen^6^; reicht eine ambulante Therapie aus, so hat die Krankenkasse die Kosten eines Krankenhausaufenthaltes nicht zu tragen. Eine Begrenzung dieses Grundsatzes im Hinblick auf bestimmte Indikationen und/oder Methoden existiert nicht, das Nachrangverhältnis der stationären Leistungserbringung gilt mithin auch uneingeschränkt bei der Implantation von implantierbaren Defibrillatoren und Geräten insbesondere zur kardialen Resynchronisation. Eine Ausnahme bilden soziale bzw. sozialmedizinische Aspekte, die die medizinische Behandlung direkt beeinflussen könnten. Diese Aspekte müssen für jeden Patienten individuell abgewogen werden. In Anlehnung an die Kriterien des „German Appropriateness Evaluation Protocol“ führen Schumacher et al. [1] eine fehlende Kommunikations- oder Transportmöglichkeit bzw. eine schlechte Erreichbarkeit durch notfallleistende Stellen, die mangelnde Einsichtsfähigkeit des Patienten oder fehlende Versorgungsmöglichkeiten als Anhaltspukte auf. Diese sollten bei der Entscheidung für eine stationäre bzw. ambulante Operation in Erwägung gezogen werden.

In der Summe ist es wichtig, eine möglichst patientenorientierte Entscheidung herbeizuführen.

### Wirtschaftlichkeit (Wahl der kostengünstigeren Alternative)

Unabhängig von der vorliegenden Evidenz müssen Leistungen in der gesetzlichen Krankenversicherung dem Wirtschaftlichkeitsgebot nach § 12 Abs. 1 SGB V entsprechen. Nach dieser Vorschrift müssen die Leistungen ausreichend, zweckmäßig und wirtschaftlich sein; sie dürfen das Maß des Notwendigen nicht überschreiten. Leistungen, die nicht notwendig oder unwirtschaftlich sind, können Versicherte nicht beanspruchen, dürfen die Leistungserbringer nicht bewirken und die Krankenkassen nicht bewilligen. Dies gilt für alle Leistungsbereiche des SGB V; mithin auch für Leistungen in Krankenhäusern.^7^ Entsprechend des Minimalprinzips sind Krankenhäuser bei medizinisch gleichwertigen Therapieansätzen verpflichtet, den kostengünstigeren zu wählen^8^; mithin auch auf eine ambulante Therapie auszuweichen. Sofern zwei verschiedene Behandlungsalternativen existieren, die gleich zweckmäßig und gleich notwendig sind, dürfen die Kosten für den gleichen zu erwartenden Erfolg nicht höher sein^9^, und es ist die kostengünstigere Leistung zu wählen. Bei der medizinischen Gleichwertigkeit der Implantation von ICDs, CRTs und ILR im Krankenhaus im Vergleich zur Implantation im niedergelassenen Bereich bedeutet dies, dass die ambulante Implantation durch einen Vertragsarzt dem Maß des Notwendigen nach § 12 Abs. 1 S. 1 SGB V entspricht. Allgemein ausgedrückt folgt hieraus Folgendes: Fehlt es an der medizinischen Erforderlichkeit einer stationären Krankenhausbehandlung, so besteht kein Vergütungsanspruch, wenn ein Krankenhaus dennoch stationär behandelt.^10^

### Abgrenzung ambulant zu stationär

Nach der Rechtsprechung findet eine Behandlung dann ambulant statt, wenn der Patient die Nacht vor und die Nacht nach dem Eingriff nicht im Krankenhaus verbringt. Demgegenüber müssen für eine stationäre Behandlung zwei Voraussetzungen erfüllt sein:

1. die Aufnahme des Patienten durch den Krankenhausarzt,
2. eine bestimmte Aufenthaltsdauer.

Vor der Aufnahme des ambulanten Operierens im Krankenhaus in das SGB V war lediglich die Aufnahme entscheidend, gemeint als „physische und organisatorische Eingliederung des Patienten in das spezifische Versorgungssystem des Krankenhauses“.^11^ Wegen der Verbreitung des ambulanten Operierens durch niedergelassene Ärzte und der Einführung dieser Behandlungsmöglichkeit in Krankenhäusern genügt die Aufnahme allein nicht mehr als Kriterium, um eine klare Abgrenzung von der ambulanten Behandlung zu ermöglichen.^12^

## Bisherige Möglichkeiten der ambulanten Implantation von CIEDs in Deutschland

Aktuell bestehen in Deutschland zwei generelle Möglichkeiten zur ambulanten Implantation von Herzschrittmachern, Defibrillatoren oder CRT-Systemen: Verträge zur besonderen Versorgung nach § 140 a SGB V oder Verträge nach § 115 b SGB V – „Ambulantes Operieren und sonstige stationsersetzende Eingriffe im Krankenhaus“. Alte Verträge zur besonderen ambulanten Versorgung nach § 73 c SGB V werden durch die Neuregelung des § 140 a SGB V abgedeckt und genießen Bestandschutz.

Der § 140 a SGB V ermöglicht sektoren- und disziplinenübergreifende Versorgungsmodelle (integrierte Versorgung) in der gesetzlichen Krankenversicherung, die eine bedarfsgerechte und effiziente Versorgung sicherstellen sollen. Nach umfassenden Recherchen der Autoren werden aktuell die in Tab. 1 aufgeführten Verträge angeboten.

**Table 1:** 

Bundesland	Vertrag/Managementgesellschaft und Krankenkassen	Inhalte
Bundesweit	MED Management GmbH Techniker Krankenkassen, DAK-Gesundheit	Enthalten sind: – Ambulante Implantationen eines ICDs oder Herzschrittmachers inkl. CRT, Systeme zur kardialen Kontraktionsmodulation (CCM) und unter bestimmten Bedingungen ILR – Ausgewählte Device-Generationen aller Hersteller sind spezifisch im Preis mit dem Vertrag verhandelt (zwischen den Herstellern und der Management-Gesellschaft) – Telemedizinische Leistungen, wie das Abfragen und Monitoring der Devices Unabhängige, externe, begleitende Maßnahme zur Qualitätssicherung von Operationen im ambulanten Bereich: DOQUVIDE – *Dokumentation der Qualität bei der Erhebung von Vitalparametern durch implantierte Devices* in Kooperation mit der Deutschen Stiftung für chronisch Kranke, die nicht Bestandteil vom BV-Vertrag ist
Bundesweit	REBECA BKK Mobil Oil BKK Novitas VAG Hessen (ca. 51 BKKen)	Implantation – Herzschrittmacher – Eventrekorder (nur VAG Hessen, BKK Mobil Oil) – ICD und CRT (nur VAG Hessen)
Bundesweit	MICADO 46 BKKen	Implantation – Herzschrittmacher – ICD – Eventrekorder
Bayern, NRW	REBECA	Implantation – Herzschrittmacher
Baden-Württemberg	MEDI Baden-Württemberg e. V. AOK Ba-Wü Bosch BKK, VAG BKK	Implantation – Neuimplantationen von 1‑ oder 2‑Kammer-Systemen (Herzschrittmacher und ICD) – Aggregatwechsel von 1‑, 2‑ oder 3‑Kammer-Systemen (Herzschrittmacher und ICD) – Ambulante Implantation von ILR Implantationspauschale (inkl. prä- und postoperativer Leistungen, Anästhesie, aller Sachkosten), Hohe Qualitätskriterien sind im Vertrag enthalten
Hessen	AOK	Implantation und Wechsel – 1-und 2‑Kammer-Herzschrittmacher

Einen weiteren Ansatz zur Verzahnung der niedergelassenen Vertragsärzte und der stationären Versorgung bietet der § 115 b SGB V – Ambulantes Operieren und stationsersetzende Eingriffe im Krankenhaus (AOP-Vertrag). Neben der Verknüpfung der ambulanten und stationären Versorgung ist es Ziel, „einheitliche Rahmenbedingungen zur Durchführung ambulanter und sonstiger stationsersetzender Eingriffe im niedergelassenen Bereich und im Krankenhaus zu schaffen“. Die Deutsche Krankenhausgesellschaft, die Kassenärztliche Bundesvereinigung und der GKV-Spitzenverband haben auf Basis des § 115 b SGB V den sog. AOP-Vertrag geschlossen. Dieser beschreibt verschiedene Kooperationsarten im Rahmen des ambulanten Operierens:

1. Der Operateur und der Anästhesist sind Mitarbeiter des Krankenhauses.
2. Die Operation wird durch einen Belegarzt durchgeführt. Dieser ist nicht am Krankenhaus angestellt, kann aber die Infrastruktur des Krankenhauses nutzen.
3. Es besteht eine vertragliche Zusammenarbeit zwischen dem Krankenhaus und einem niedergelassenen Vertragsarzt. Durch diesen wird die ambulante Operation durchgeführt (s. AOP-Vertrag § 7 Abs. 4).
4. Die Eingriffe erfolgen in einer rein ambulanten Versorgungsstruktur (z. B. ambulantes Operationszentrum oder Praxis des Vertragsarztes).

Für die Zulassung ambulanter Operationen muss das Krankenhaus den zuständigen Landesverbänden der Krankenkassen und den Verbänden der Ersatzkassen, der Kassenärztlichen Vereinigung sowie dem Zulassungsausschuss eine maschinenlesbare Mitteilung übersenden (§ 1 AOP-Vertrag). Vertragsärzte benötigen eine Genehmigung der jeweiligen Kassenärztlichen Vereinigung, um ambulante Operationen durchführen zu dürfen.

Als Abrechnungsgrundlage für das ambulante Operieren gilt der Einheitliche Bewertungsmaßstab (EBM), insbesondere Kap. 31. Die Abrechnungsgrundlage von ambulanten Operationen ist für das Krankenhaus und den niedergelassenen Bereich grundsätzlich gleich. Die AOP-Vergütungen unterliegen keinen Mengenbegrenzungen und werden außerhalb des Budgets bezahlt. Der AOP-Katalog definiert alle ambulant zu erbringenden und abrechenbaren Leistungen. In der Regel wird der Leistungskatalog jährlich neu verhandelt. Die einzelnen Leistungen im AOP-Katalog werden durch OPS-Codes definiert und mit einem OPS-Text beschrieben, z. B. OPS-Code 5‑377.1: Implantation eines Herzschrittmachers, Defibrillators und Ereignisrekorders: Schrittmacher, 1‑Kammer-System. Zudem werden jeder Leistung Kategorien zugeordnet. Leistungen der Kategorie 1 sollen in der Regel ambulant, die der Kategorie 2 ambulant oder stationär durchgeführt werden. Die stationäre Aufnahme muss in dem Fall gegenüber der Krankenkasse begründet werden.

Die Höhe der Vergütung richtet sich nach dem EBM, z. B. operativer Einbau/Aggregatwechsel (EBM-Ziffern 31211, 31212, 31214) und postoperative Überwachung (EBM-Ziffer 31503), dazu kommt ggf. die Überwachung der Vitalfunktion (EBM-Ziffer 05340) und die Durchleuchtung (EBM-Ziffer 34280). Wird die Leistung durch das Krankenhaus erbracht, erfolgt die Abrechnung der Leistung direkt mit der Krankenkasse des Patienten. Ist ein Belegarzt der Operateur, gibt es zwei Möglichkeiten:

- Zum einen kann das Krankenhaus mit der Krankenkasse abrechnen. Der Belegarzt erhält in dem Fall die Vergütung vom Krankenhaus.
- Zum anderen kann der Belegarzt sein Honorar selbst abrechnen und dem Krankenhaus ein Nutzungsentgelt zahlen. Ist der niedergelassene Arzt vertraglich an das Krankenhaus gebunden, rechnet das Krankenhaus die Leistung ab und vergütet den Arzt je nach individuellem Vertragsinhalt. Für das Aggregat-Sondensystem gilt eine gesonderte Abrechnung. In den meisten Bundesländern erfolgt diese direkt zwischen dem Hersteller oder Lieferanten des Systems und der Krankenkasse (s. unten).

Der AOP-Vertrag regelt zudem prä-, intra- und postoperative Leistungen (§ 4–6 AOP-Vertrag). Als postoperative Leistungen werden Leistungen verstanden, die den Behandlungserfolg sicherstellen, wie erneute Device-Abfragen, Wundkontrollen und andere Tätigkeiten (Kontrolle Medikation etc.).

Nach §‑115 b SGB V sind Qualitätssicherungsmaßnahmen im Sinne des § 135 Abs. 2 SGB V zu vereinbaren. Hier sind fachliche, organisatorische, hygienische, räumliche und apparativ-technische Voraussetzung zu definieren.

Problematisch ist, dass nur Leistungen abgerechnet werden können, die für Herzschrittmachereingriffe (1- und 2‑Kammer-Aggregate) gelten. CRT- (CRT‑P und CRT-D), ICD- und ILR-Eingriffe sind aktuell nicht Gegenstand des ambulanten Operierens. Ein signifikanter Anteil von Eingriffen bei Patienten mit CIED, die unter medizinischen/organisatorischen Gesichtspunkten problemlos ambulant erbracht werden können, ist daher im Rahmen des ambulanten Operierens nicht abrechenbar.

Zudem kann unter der gegebenen Kostenerstattung für 2‑Kammer-Herzschrittmacherimplantation (55 min Schnitt/Hautnaht ca. 520 €) mit einer einfachen Prozesskostenrechnung nicht kostendeckend gearbeitet werden. Hinzuzuziehen sind Aufwände für die Vor- und Nachbereitung der Räumlichkeiten unter Hygienestandards (ca. 30–45 min), Personalkosten für mindestens 2 Assistenzpersonen (MFA/Krankenpflege), Sachkosten (abgesehen vom Device), Raummieten sowie Kosten für die Durchleuchtungsanlage (DL-Anlage). Die Kostenerstattung für 1‑Kammer-Herzschrittmacherimplantationen enthält für 35 min Schnitt/Hautnaht ca. 375 €; für einen Aggregatwechsel: Schnitt/Hautnaht 15–30 min, ca. 235 €.

Da die Leistung im niedergelassenen Bereich erbracht wird, müssen Anästhesieleistungen auch ggf. über niedergelassene Anästhesisten abgerechnet werden (sofern diese überhaupt in Anspruch genommen werden). Von Kardiologen erbrachte Kardioanalgosedierungsleistungen sind nicht verhandelt, demnach auch nicht abrechenbar (es existiert keine OPS-Ziffer). Sie sollten unbedingt Gegenstand weiterer Verhandlungen darstellen.

Das Verfahren zur Kostenerstattung ist sehr heterogen gestaltet. Während die ärztliche und technische Leistung einheitlich über die KV abgerechnet wird, erfolgt die Abrechnung der Aggregate und Sonden von Bundesland zu Bundesland unterschiedlich – teilweise über Rezept, teilweise als Rechnungsweitergabe an die KV mit der Abrechnung im Quartal unter Sachmitteln und einer Bezahlung nach 6 Monaten, teilweise als Pauschale. Problematisch erscheint zum einen auch die Tatsache, dass keine Qualitätsvereinbarungen, z. B. über Standards und die Ausstattung der Räumlichkeiten, bestehen. Zum anderen werden Sachkosten, z. B. für OP-Materialien analog zur ambulanten Herzkatheteruntersuchung, nicht vergütet. Wie bereits bei AOP-Leistungen im Krankenhaus, kann mit einer einfachen Prozesskostenrechnung keine Kostendeckung erzielt werden. Hinzu kommen länderunterschiedliche Vergütungen, da ggf. unterschiedliche Punktwerte einfließen.

Zusammenfassend kann demnach festgehalten werden, dass aktuell einige Verträge nach § 140 a SGB V existieren, die teilweise hohe Qualitätsanforderungen an strukturell-organisatorische Gegebenheiten und die Ärzte stellen. Andererseits können über den § 115 b SGB V ambulante Eingriffe nur bei 1‑ und 2‑Kammer-Schrittmachern erfolgen. Für ICD- und CRT-Systeme sowie für Ereignisrekorder bestehen hier keine Möglichkeiten der ambulanten Leistungserbringung. Weiterhin fehlt es in diesem Zusammenhang an verbindlichen, standardisierten Qualitätskriterien und einer Qualitätssicherung. Auch eine Auswertung dieser Daten im Sinne der Weiterentwicklung des Systems ist derzeit nicht implementiert. Darüber hinaus erschweren die Heterogenität der Abrechnungsverfahren und die teilweise unzureichenden Vergütungspauschalen die qualitativ hochwertige Bereitstellung der Leistung.

Die dargestellte sehr heterogene Sachlage erschwert oder verhindert in weiten Teilen die ambulante Erbringung einer Gruppe operativer Leistungen bei ICD- und CRT-Systemen sowie bei implantierbaren Ereignisrekordern, welche aus medizinischer Sicht in vielen Fällen sinnvoll und qualitativ hochwertig als ambulante Leistung durchgeführt werden könnte.

## Stand der Wissenschaft zum Nutzen ambulanter Implantationen

Die Notwendigkeit einer patientenzentrierten Versorgung und Behandlung von Patienten mit CIEDs rückte in den letzten Jahren immer mehr in den Fokus. Nachdem durch zahlreiche technische Innovationen und neue wissenschaftliche Studienergebnisse erhebliche Fortschritte erzielt wurden, erscheint es nun mehr als eine *Conditio sine qua non*, aus der Vielfalt von Optionen die richtige für den jeweiligen Patienten zu selektieren. Dies kommt auch deutlich in der von der European Heart Rhythm Society (EHRA) herausgegebenen Stellungnahme/Positionspapier 2019 zum Ausdruck, in der das Fehlen von personalisierten und individualisierten Ansätzen bemängelt wird. Das als „gap“ bezeichnete Fehlen solcher Ansätze finden sich nicht nur in der interventionellen Elektrophysiologie, sondern auch auf dem Sektor der Device-Implantationen [3].

Im Folgenden sollen kurz beispielhaft Ergebnisse wissenschaftlicher Untersuchungen dargestellt werden, welche die Sicherheit und den Nutzen der ambulanten Implantation verdeutlichen.

**Figure 1:**
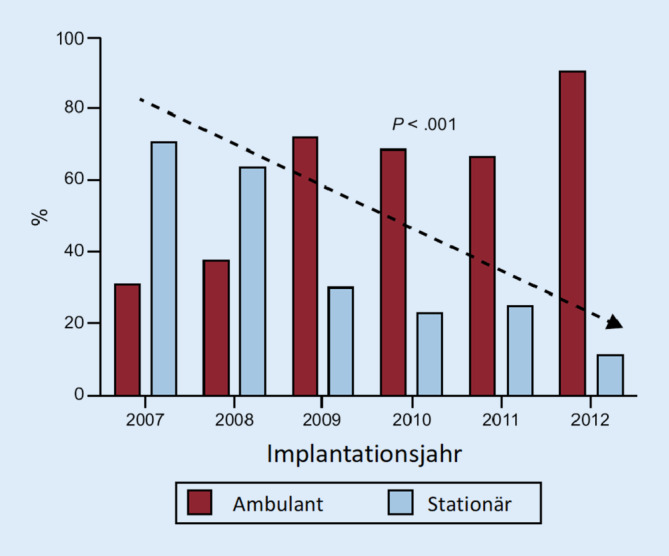

Bereits 2015 wurde in Spanien die Machbarkeit, die Sicherheit und Kosteneffizienz einer ambulanten Operation von implantierbaren ICDs untersucht und belegt [4]. An einem Kollektiv von über 400 Implantationen während der Studiendauer von 5 Jahren wurden primär 58 % der Patienten ambulant versorgt. Von diesen Patienten mussten knapp 10 % nach der Operation stationär aufgenommen werden. Gründe für die Aufnahme beruhten überwiegend auf den Entscheidungen der ambulanten Behandler. Hinzu kamen vereinzelte relevante Taschenhämatome sowie soziale Umstände. Eingeschlossen in diese Erhebung wurden Patienten, welche zum ICD-Wechsel oder zur Neuimplantation bei Primärprävention anstanden. Die Grafik zeigt das Verhältnis von ambulanter zu stationärer Operation auf. Hieraus geht deutlich die Lernkurve über die Zeitdauer hervor (Abb. 1).

Im Jahr 2016 wurden im Rahmen der Jahrestagung der Heart Rhythm Society die Ergebnisse der „Same-day discharge for ICD implant“ vorgestellt. Als primären Endpunkt wählten die Investigatoren die Komplikationsrate innerhalb der ersten 30 Tage nach Operation. Eingeschlossen wurden Patienten mit Primärprävention. Bei den nahezu 600 eingeschlossenen Patienten zeigte sich eine Nichtunterlegenheit zur stationären Implantation.^13^ Wesentliche Säulen stellten das Remote-Monitoring sowie die spezifische Patientenauswahl, also die individuelle Anpassung der Therapie, dar.

**Figure 2:**
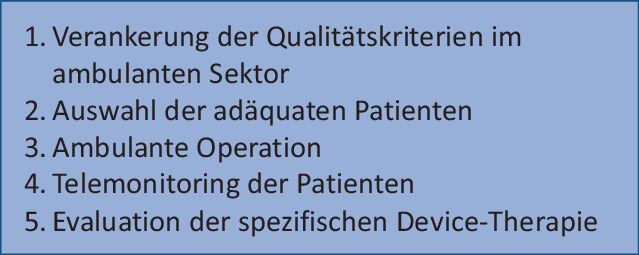

Es zeigt sich, dass es immer noch an einem holistischen Ansatz der personalisierten Medizin in der Device-Therapie mangelt. Ein Teil dieses individualisierten Ansatzes stellt sicherlich die Verlagerung der Implantationen der CIEDs in den ambulanten Bereich dar. Wichtig hierbei sind die richtige Patientenauswahl, die genaue Evaluation der spezifischen Device-Behandlung sowie die Ausstattung mit Telemonitoring als integralen Bestandteil (Abb. 2).

## Ganzheitliches Qualitätskonzept zur ambulanten Implantation von CIEDs

Die vorangehenden Ausführungen beschreiben die Notwendigkeit einer individualisierten Verlagerung der Device-Therapie in den ambulanten Bereich. Dies leitet sich aus einer zunehmend personalisierten Versorgung der Patienten, aus dem Grundsatz „ambulant vor stationär“ sowie dem Wirtschaftlichkeitsgebot ab. Aktuelle Möglichkeiten der ambulanten Implantation legen jedoch unterschiedliche Standards an strukturelle, organisatorische und kompetenzspezifische Kriterien zu Grunde. Es existiert kein einheitlicher Qualitätsstandard. Um die Qualität zu sichern und die Vergleichbarkeit der Leistungen zu ermöglichen, bedarf es eines ganzheitlichen Qualitätskonzeptes zur ambulanten Implantation von CIEDs, das im Folgenden vorgestellt wird.

### Idee

**Figure 3:**
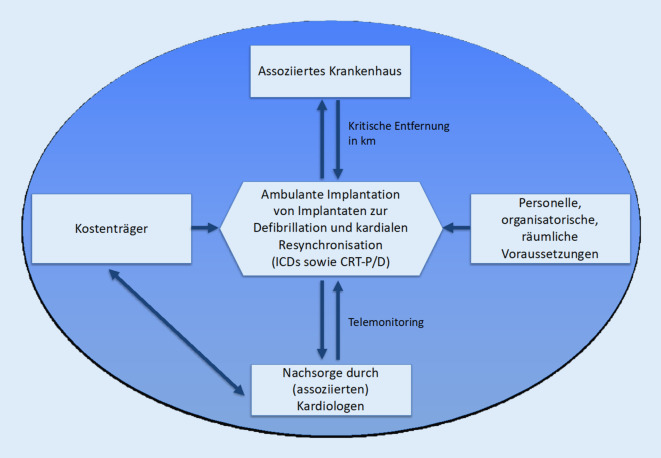

Das Qualitätskonzept beinhaltet die Betrachtung der personellen, organisatorischen und räumlichen Voraussetzungen für ambulante Operationen. Zudem erfolgt die Personalisierung der Leistung durch eine individuelle Auswahl der Patienten nach geeigneten Kriterien. Für die Nachsorge der Patienten bietet sich ein Telemonitoring-Ansatz an, der durch den nachsorgenden Kardiologen koordiniert wird. Letztlich sind sowohl ein assoziiertes Krankenhaus als auch die Kostenträger Teil des Konzeptes. In Abb. 3 wird eine Übersicht über das Konzept gegeben.

### Personelle Voraussetzungen

Die ambulante Implantation und der Aggregatwechsel bei ICD- und CRT-Systemen sowie ILR stellt besondere Anforderungen an Ärzte, nichtärztliches Personal und an die Einrichtung, in welcher die Eingriffe durchgeführt werden sollen.

Es müssen mindestens 3 Personen (Operateur, Instrumentenpfleger und unsterile Hilfsperson) eingeplant werden. Bei Risikopatienten muss darüber hinaus ein in der Notfall- und/oder Intensivmedizin erfahrener Arzt die Überwachung übernehmen. Die Anwesenheit eines Facharztes für Anästhesie ist zudem bei Eingriffen, die in Intubationsnarkose geplant sind, selbstverständlich. Bei allen anderen Konstellationen ist dies fakultativer Natur und kann selbstverständlich auch durch den in Notfall- oder Intensivmedizin erfahrenen Kardiologen bewerkstelligt werden.

Anforderungen an den Implanteur:

- Facharzt für Innere Medizin mit der Zusatzbezeichnung Kardiologie oder dem Facharzt für Herzchirurgie
- Zusatzqualifikation spezielle Rhythmologie „Aktive Herzrhythmusimplantate“ der Deutschen Gesellschaft für Kardiologie – Herz- und Kreislaufforschung e. V.

*… oder* (in Anlehnung an [5, 6]):

- Mindestens 75 transvenöse Schrittmacherimplantationen als primärer Operateur unter Anleitung eines erfahrenen Ausbilders, darunter mindestens 50 % 2‑Kammer-Systeme und 25 Aggregatwechsel bzw. Revisionseingriffe
- 25 ICD- und 10 CRT-Implantationen als primärer Operateur (initial unter Supervision) und 10 Aggregatwechsel bzw. Revisionen von ICD- oder CRT-Systemen
- Mindestens 250 Schrittmacher-, einschließlich Programmierung des Systems, davon mindestens die Hälfte bei 2‑Kammer- und frequenzadaptiven Systemen, 50 ICD- und 30 CRT-Kontrollen selbstständig durchgeführt

*… zusätzlich:*

- Nachweis einer kontinuierlichen Implantation von ICD- oder CRT-Systemen in den 3 Jahren vor Aufnahme der ambulanten Implantationstätigkeit.

### Organisatorische Voraussetzungen

Vor dem Eingriff müssen mindestens die folgenden Dokumente vorliegen:

- Unterschriebene Einverständniserklärung
- Checkliste für den geplanten Eingriff
- Relevante medizinische Vorbefunde
- Aktueller Medikationsplan
- Aktuelle Laboruntersuchung, inkl. kleinem Blutbild, Gerinnungsparameter, CRP, Nierenfunktionsparameter (Serum-Kreatinin, GFR) sofern ein Kontrastmittel verwendet wird.

Nach der Implantation muss die Überwachung des Patienten durch geschultes Personal über mindestens 2 Stunden sichergestellt sein. Es muss eine adäquate Dokumentation über den erfolgten Eingriff und die gewählten Implantate erfolgen. Diese enthält alle wichtigen Informationen und muss dem nachbehandelnden Arzt zugänglich gemacht werden.

Es muss sichergestellt werden, dass bei Komplikationen ein Revisionseingriff notfallmäßig durchführbar ist. Dies kann durch den Operateur selbst, eine Bereitschaftsdienstregelung oder eine Kooperation mit einer stationären Einrichtung realisiert werden. Zudem muss auch sichergestellt sein, dass bei intraoperativen Komplikationen kompetente, fachübergreifende Unterstützung kurzfristig verfügbar ist.

Eine korrekte Lage des Aggregats und der Sonden muss radiologisch dokumentiert werden.

Vor Entlassung des Patienten müssen ein Perikarderguss und ein Pneumothorax sicher ausgeschlossen sein. Daher gilt eine sonographische Kontrolle des Perikards als obligat. Die Möglichkeit zur Anfertigung eines Röntgen-Thorax vor Entlassung sollte gegeben sein, sofern dies klinisch indiziert ist.

Die Wundkontrolle und Überprüfung der Systemintegrität werden bei Vorstellung am nächsten Tag durchgeführt. Hierzu zählen eine komplette Kontrolle und ggf. eine Änderung der Programmierung des CIED.

Letztlich muss der Patient über Möglichkeiten der Kontaktaufnahme nach dem Eingriff im Notfall (Notfalltelefon) schriftlich informiert sein.

Die Teilnahme an einer sektorenübergreifenden Qualitätssicherung sollte obligat sein, sobald diese verfügbar ist.

### Räumliche/hygienische Voraussetzungen

Der bauliche, funktionelle, raumlufttechnische und hygienische Standard eines Operationsraums oder eines entsprechend ausgestatteten Herzkatheterlabors muss auch im Kontext der ambulanten Operation unbedingt gegeben sein. Dies gilt analog zu den im stationären Bereich geforderten Voraussetzungen. Zudem muss eine Durchleuchtungsmöglichkeit in verschiedenen Ebenen vorhanden sein. Darüber hinaus gelten die im Strukturpapier der DGK geforderten Voraussetzungen [6].

### Selektionsmerkmale der Patienten

Prinzipiell ist es natürlich möglich, den operativen Eingriff für die Implantation des elektrischen Geräts bei allen Patienten ambulant zu gestalten. Jedoch sollte dies bei einigen Patienten mit Vorsicht bedacht werden.

Hierzu zählen im Besonderen folgende Parameter vor dem operativen Eingriff:

- komplexe angeborene Herzfehler,
- schwerwiegende Herz-Kreislauf-Erkrankungen, z. B. NYHA III–IV,
- respiratorische Partialinsuffizienz mit Notwendigkeit zur Sauerstofftherapie oder respiratorische Globalinsuffizienz,
- soziale bzw. sozialmedizinische Aspekte, die eine Auswirkung auf die medizinische Behandlung haben könnten, z. B. keine Kommunikationsmöglichkeit, keine Transportmöglichkeit oder schlechte Erreichbarkeit durch Stellen, die Notfallhilfe leisten können, mangelnde Einsichtsfähigkeit des Patienten, fehlende Versorgungsmöglichkeiten (in Anlehnung an German Appropriateness Evaluation Protocol – G-AEP [1]).

Während des Eingriffs können gewisse Ereignisse die Charakteristik verändern. Je nach Veränderung muss dann der Operateur die Entscheidung entsprechend fällen. Hierzu können folgende Parameter exemplarisch auftreten:

- schwieriger intraoperativer Verlauf, z. B. dokumentierte arterielle Fehlpunktionen, Verdacht auf Verletzung der Lunge oder neu aufgetretenem Perikarderguss mit Notwendigkeit stationärer Kontrollen,
- schwierige Sondenplatzierung (mehr als 10 dokumentierte Umpositionierungen einschließlich Operationsdauer ≥ 60 min beim 1‑Kammer-System und ≥ 120 min beim 2‑Kammer-System) mit Gefahr frühpostoperativer Sondendislokation,
- unzureichend kontrollierte arterielle Hypertonie mit wiederholt dokumentierten Werten > 180 mm Hg systolisch und/oder diastolisch > 110 mm Hg,
- durch orale Therapie nicht ausreichend kontrollierte starke Wundschmerzen mit Notwendigkeit zur parenteralen Schmerztherapie,
- relevante Nachblutungen mit Notwendigkeit zusätzlicher therapeutischer Maßnahmen (z. B. zusätzliche Wundkompression, Revisionsoperation, Redondrainage, Transfusion).

### Assoziierte Kardiologen/Krankenhäuser

Wie oben bereits in den organisatorischen Voraussetzungen festgehalten, ist in Notfällen die Kooperation mit einem assoziierten Krankenhaus unbedingt erforderlich und im Vorfeld am besten vertraglich abzusichern. Darüber hinaus muss der Übergang des Patienten zu dem nachsorgenden Kardiologen so gestaltet werden, dass diesem alle wichtigen Informationen vorliegen und die Nachbetreuung des Patienten reibungslos erfolgen kann. Hierzu eigenen sich natürlich der gemeinsame Zugriff auf internetbasierte Datenbanken.

### Dezidierte Vergütungskonzepte

Es kommen die 2 Möglichkeiten der Vergütung infrage, die auch schon in den bestehenden Verträgen existieren:

1. eine Pauschalvergütung für peri- und intraoperative Leistungen, inkl. aller Material‑, Aggregat- und Sondenkosten (wie z. B. im Facharztvertrag Kardiologie in Baden-Württemberg), oder
2. eine pauschale Vergütung für die Implantationsleistung (Personalkosten, Sachkosten für Materialen der Operation, Raumkosten, Hygieneaufwand, Raummiete, Durchleuchtung) in Anlehnung an den Vertrag mit der MED Management GmbH. Das Aggregat und Sonden werden extra vergütet. Dazu sollte ein Katalog mit dezidierten Preisen mit regelmäßiger Aktualisierung bestehen. Die Aktualisierung sollte dem Fortschritt der technischen Entwicklungen Rechnung tragen.

### Nutzen der Telemedizin als integralem Bestandteil

Insbesondere mit Blick auf die ambulante Implantation von kardialen Devices bietet die Telemedizin große Potenziale [7]. Einerseits ermöglicht sie durch ein begleitendes Monitoring und integrierte Betreuungskonzepte die frühe Identifikation einer drohenden Dekompensation des Patienten. Andererseits kann das begleitende Telemonitoring die Abstimmung zwischen Haus- und Fachärzten sowie Krankenhäusern erleichtern. Durch den vereinfachten Informationsaustausch können potenzielle Ineffizienzen zwischen den Leistungserbringern [8] verringert werden.

Wichtig ist, dass auf die Datenübertragung eine stringente Handlungskonsequenz folgt. Fehlt diese, sind keine signifikanten Verbesserungen im klinischen Verlauf festgestellt worden [9, 10]. Die Arbeitsgruppe 33 Telemonitoring der Deutschen Gesellschaft für Kardiologie, Herz- und Kreislaufforschung e. V. hat hierzu konkrete Handlungsempfehlungen erarbeitet [11]. Diese Empfehlungen für standardisierte Handlungskonsequenzen beinhalten beispielsweise bei einer Herzrhythmusstörung (ICD-Schock) eine unmittelbare Kontaktaufnahme mit dem Patienten und die Einbestellung des Patienten zur Kontrolle, wobei der Zeitplan dem klinischen Zustand des Patienten angepasst werden kann.

Solche Voraussetzungen sollten natürlich alle Kooperationspartner aufweisen können, um eine möglichst optimale, individualisierte Behandlung und Nachsorge realisieren zu können.

## Fazit und Perspektiven

Der Artikel zeigt die Notwendigkeiten und die konsekutive Machbarkeit für ambulante, operative Eingriffe für Patienten mit CIEDs klar auf. Das entwickelte Qualitätskonzept dient als Grundlage, um einen sektorenübergreifenden, fachspezifischen Diskus anzuregen und die ambulante Operation nachhaltig im deutschen Gesundheitsmarkt zu etablieren. Parallel müssen gemeinsam mit den Kostenträgern für beide Seiten attraktive Finanzierungsmodelle erarbeitet werden.

Operative Eingriffe müssen in der heutigen Zeit nicht zwingend einen längeren Krankenhausaufenthalt mit sich bringen. Die bis dato erreichten Fortschritte müssen durch ihre Einführung in die Versorgungsprozesse neue hochwertige Standards erzeugen, welche durch klar definierte Qualitätssicherungsmaßnahmen begleitet werden. Erst hierdurch werden für ein klar definiertes Patientenkollektiv Vorteile generiert. Sekundär kommt es zu Kosteneinsparungen im Gesundheitssystem. Diese Vorgehensweise wird durch die Implementation der Telemedizin begleitet, um so eine optimale, individuelle Versorgung des Patienten zu erzeugen.

Perspektivisch stellt die ambulante Implantation der CIEDs die Voraussetzung dar, um in den kommenden Jahren eine noch stärker an den Bedürfnissen der Patienten ausgerichtete Versorgung zu ermöglichen. Mit der Hilfe der sich zusehends etablierenden Möglichkeiten der künstlichen Intelligenz (KI) lassen sich diese Prozesse auf höchstem medizinischem Niveau weiter etablieren [12, 13]. KI und Telemedizin werden dadurch von zentraler Bedeutung für die ambulante Versorgung schwer kranker Patienten.

## Footnotes

1 Auch bei privaten Krankenversicherungen gilt der Vorrang der ambulanten vor der stationären Heilbehandlung, ohne dass es eine gesetzliche Normierung im Versicherungsvertragsgesetz gibt, zuletzt Urteil des LG Mannheim vom 10. September 2020, Az. 9 O 383/19.

2 Vgl. auch Gamperl, in KassKomm, SGB V, § 39, Rn. 47.

3 Ständige höchstrichterliche Rechtsprechung, vgl. bspw. Urteil des Bundessozialgerichts (BSG) vom 16. Februar 2005, Az. B 1 KR 18/03 R.

4 Vgl. Bundestagsdrucksache 12/3608, S. 103.

5 Vgl. Bundestagsdrucksache 11/2237, S. 177.

6 Urteil des BSG vom 17. November 2015, Az. B 1 KR 20/15 R.

7 Zuletzt Urteil des BSG vom 1. Juli 2014, Az. B 1 KR 62/12 R.

8 Beschluss des BSG vom 31. Mai 2006, Az. B 6 KA 68/05 B.

9 Urteil des BSG vom 7. Mai 2013, Az. B 1 KR 53/12 R.

10 Urteil des BSG vom 17. November 2015, Az. B 1 KR 20/15 R; nach den Ausführungen des 1. Senates im selben Urteil bedeutet Krankenhausbehandlungsbedürftigkeit einen Krankheitszustand, dessen Behandlung den Einsatz der besonderen Mittel eines Krankenhauses erforderlich macht.

11 Bundestagsdrucksache 12/3608, S. 82.

12 Urteil des BSG vom 28. Februar 2007, Az. B 3 KR 17/06 R.

13 https://www.medscape.com/viewcollection/33690.
